# The Usefulness of Cardiopulmonary Exercise Testing to Detect Functional Improvement after Transcatheter Valve Procedures: What Do We Know So Far?

**DOI:** 10.31083/j.rcm2509336

**Published:** 2024-09-20

**Authors:** Luca Cumitini, Ailia Giubertoni, Giuseppe Patti

**Affiliations:** ^1^Department of Translational Medicine, University of Eastern Piedmont, 28100 Novara, Italy; ^2^Division of Cardiology, Maggiore della Carità Hospital, 28100 Novara, Italy

**Keywords:** cardiopulmonary exercise testing, heart failure, transcatheter valve procedures

## Abstract

Transcatheter valve procedures have become a cornerstone in the management of patients with valvular heart disease and high surgical risk, especially for aortic stenosis and mitral and tricuspid regurgitation. Cardiopulmonary exercise testing (CPET) is generally considered the gold standard for objectively quantifying functional capacity, providing a comprehensive evaluation of the human body's performance, particularly in patients with heart failure (HF). Its accurate assessment is valuable for exploring the pathogenetic mechanisms implicated in HF-related functional impairment. It is also useful for objectively staging the clinical severity and the prognosis of the disease. The improvement in functional capacity after transcatheter valve procedures may be clinically relevant and may provide prognostic information, even in this setting. However, it remains to be fully determined as data on the topic are limited. This review aims to summarize the available evidence on the usefulness of CPET to assess functional improvement in patients undergoing transcatheter valve procedures.

## 1. Introduction

Valvular heart disease (VHD) is a leading cause of acute and chronic heart failure (HF) [1]. Aortic stenosis (AS) and mitral regurgitation (MR) are the main aetiologies of severe native VHD, often linked to congestive HF [2]. Severe tricuspid regurgitation (TR) associated with right-sided HF has been adequately recognized only recently [1]. Transcatheter valve repair/replacement now represents a cornerstone in managing patients with VHD and is widely performed since it can ameliorate the poor prognosis of these patients [1, 3]. Traditionally, AS, MR, and TR conditions have been treated surgically [4]. However, the choice between transcatheter valve procedures or surgery should be made using a shared decision-making approach, considering the patient’s preferences, surgical risk, and anatomical characteristics. A multidisciplinary team of interventional cardiologists, cardiothoracic surgeons, radiologists, echocardiographers, nurses, and social workers, known as the “heart team”, should discuss all these features to determine the best course of action for each patient [1]. In candidate patients for percutaneous valve repair, a pre-procedural multimodality imaging assessment, mainly including transthoracic echocardiography, transesophageal echocardiography, and computed tomography, is essential for planning the intervention and selecting the most appropriate device to guarantee optimal outcomes [5].

Cardiopulmonary exercise testing (CPET) is likely the most comprehensive full-body test, providing a complete evaluation of the human body’s performance [6]. This test has been significantly improved throughout the years, especially in patients with HF, as it provides a considerable amount of highly valuable diagnostic and prognostic information [7, 8]. Exercise intolerance is an important prognostic characteristic related to HF [9]. Thus, accurate quantification of exercise intolerance, is beneficial for exploring the pathogenetic mechanisms implicated in functional impairment and objectively staging the clinical severity of the disease [9]. Health-related quality of life questionnaires (e.g., Kansas City Cardiomyopathy Questionnaire (KCCQ)) are commonly used in HF patients, yet they are subjective to personal interpretation and do not reflect the objective clinical and pathophysiological status [10]. Basically, the two methods currently used in daily clinical practice to define the extent of exercise restriction are the 6-minute walking test (6MWT) [11] and CPET [12]. Guazzi *et al*. [13] established that, even if the 6MWT is a straightforward and well-founded first-line test to assess the exercise limitation in patients with HF, no evidence supports its use as a prognostic tool to replace CPET-derived information. The advantage of CPET is that it assesses exercise tolerance and evaluates the individual’s pathophysiological responses to the body’s increased metabolic demands by analyzing gas exchange (primarily O_2_ and CO_2_) and other ventilatory variables [14]. This technique enables clinicians to investigate the causes of dyspnoea and fatigue, accurately differentiate between cardiac and pulmonary disease, improve decision-making and outcome prediction, and objectively identify targets for therapy [15]. CPET is routinely used in the prognostic evaluation of patients with HF, where the prognostic significance of peak oxygen consumption (peak VO_2_) and the minute ventilation/carbon dioxide production (VE/VCO_2_) slope is well established [16, 17]. Furthermore, CPET has become a reproducible and safe technique [7]. Previously, standard indications of CPET did not include evaluating patients with VHD, as data remain limited [18]. However, the clinical assessment of these pathological conditions by CPET has been considered an option by expert consensus since 2016 [19]. Nevertheless, despite mounting evidence, CPET is not mentioned in the European Society of Cardiology 2021 guidelines for VHD [1].

**Fig. 1.**
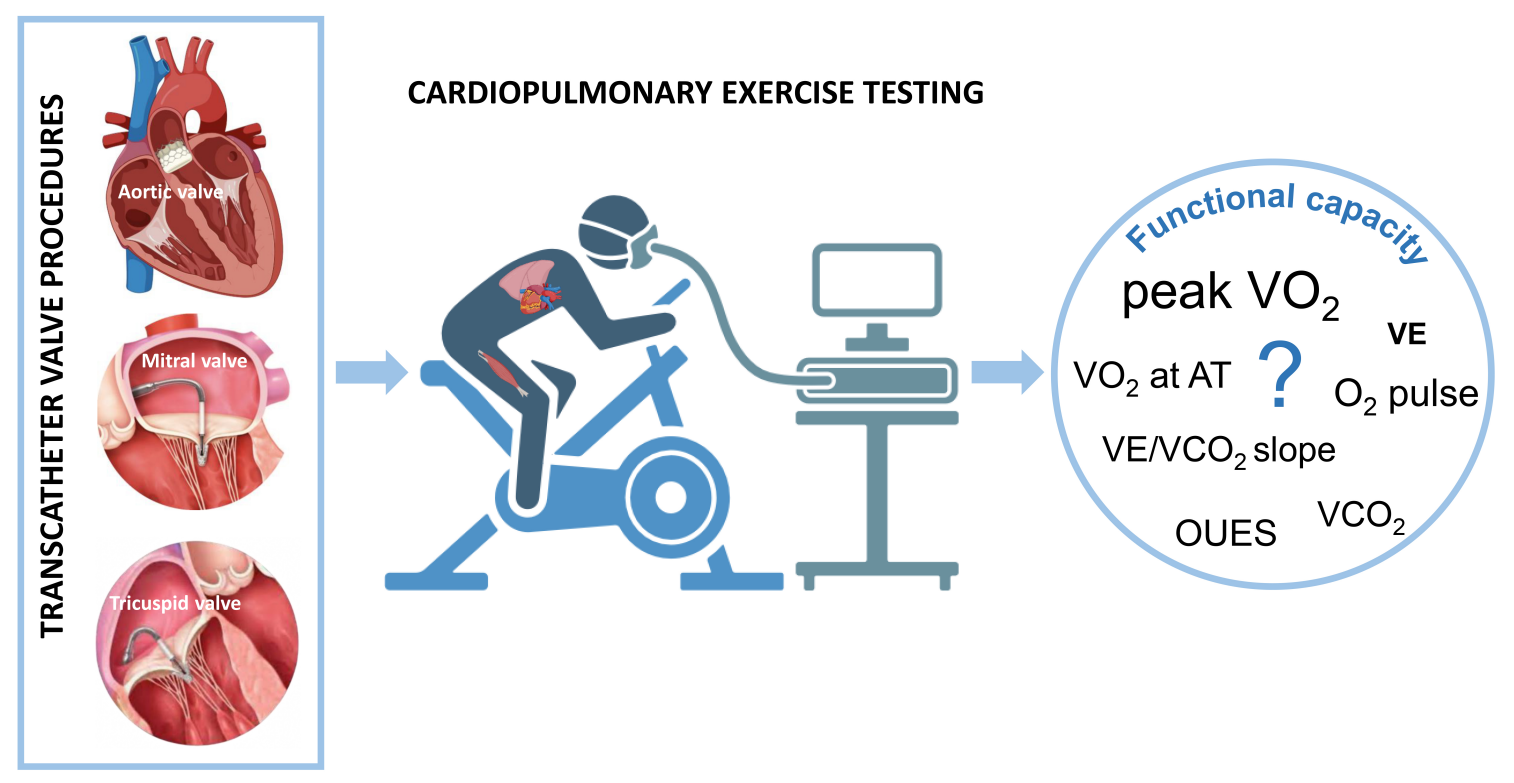
**Cardiopulmonary exercise testing in transcatheter valve procedures**. Transcatheter valve procedures have become a cornerstone in managing patients with valvular heart disease and high surgical risk. However, the objective quantification of functional improvement after procedures remains elusive due to a lack of robust data. Cardiopulmonary exercise testing, providing a comprehensive evaluation of the human body’s performance, may emerge as a promising tool in this setting. VE/VCO_2_ slope, minute ventilation/carbon dioxide production slope; VO_2_, oxygen consumption; VCO_2_, carbon dioxide production; AT, anaerobic threshold; VE, minute ventilation; OUES, oxygen uptake efficiency slope.

This review addresses such issues and attempts to summarize the evidence on the usefulness of CPET in patients undergoing transcatheter valve procedures (Fig. 1).

## 2. CPET and Transcatheter Treatment of Severe Aortic Stenosis

The recent European Valvular Heart Disease II survey showed that AS is the leading cause of single-valve disease [2]. The increase in the prevalence of this condition correlates with age, with 26.5% of AS patients being older than 80 years. Due to the unfavorable prognosis, clinical practice guidelines currently recommend early intervention in symptomatic patients with severe AS [1]. However, many patients are considered asymptomatic, and valve repair is indicated when only the left ventricular ejection fraction is reduced or conventional exercise testing cannot be tolerated [1]. An outpatient follow-up and conservative management are recommended for patients who do not meet either criteria [1]. Therefore, the decision to repair a severely stenotic aortic valve in asymptomatic patients is more complex. Transcatheter aortic valve implantation (TAVI) is an effective and safe treatment alternative for patients with severe AS, particularly if vulnerable and with multiple comorbidities. Compared to traditional surgical replacement, TAVI has indications primarily in older and frail patients with various risk factors and concomitant diseases [20]. Nevertheless, the role of TAVI in improving patients’ functional capacity has yet to be established. Murata *et al*. [21] prospectively enrolled 58 patients undergoing CPET less than 1 month after successful TAVI. Patients were followed for >1 year (median 19 months) after CPET to account for any death or HF hospitalization event. During follow-up, VE/VCO_2_ slope and minimum VE/VCO_2_ were the only significant predictors of future mortality or HF events in the univariate analysis, remaining independent predictors even after adjustment for potential confounders, including age, gender, Society of Thoracic Surgeons (STS) score, and peak VO_2_. The area under the curve (AUC) was meaningful for both VE/VCO_2_ slope (AUC = 0.734, 95% confidence interval (CI), 0.607–0.861; *p* = 0.008) and minimum VE/VCO_2_ (AUC = 0.705, 95% CI, 0.564–0.845; *p* = 0.019). Kaplan–Meier analysis revealed that a high VE/VCO_2_ slope (≥34.6) (log-rank χ^2^, 9.602; *p* < 0.01) and a high minimum VE/VCO_2_ (≥45.2) (log-rank χ^2^, 7.423; *p* < 0.01) were significantly associated with increased incidence of death and HF hospitalization during follow-up.

Generally, older patients undergoing TAVI have impaired mobility and reduced quality of life [22, 23]. Thus, the benefit of post-interventional exercise training in improving their physical capacity remains to be robustly demonstrated. Pressler *et al*. [24] reported a prospective pilot study where 27 post-TAVI patients were randomized 1:1 to an intervention group performing 8 weeks of supervised combined aerobic exercise and resistance training or usual care. The primary endpoint was the between-group difference in change of peak VO_2_, as assessed by CPET from baseline to 8 weeks. A change favoring the training group was observed, with a between-group significant difference in change of peak VO_2_ (+3.7 mL/min/kg, 95% CI, 1.1–6.3, *p* = 0.007) and oxygen uptake at anaerobic threshold (VO_2_ at AT: +3.2 mL/min/kg, 95% CI, 1.6–4.9; *p* < 0.001). Change over time in 6MWT did not differ between the two arms. These results suggest that exercise after TAVI may significantly improve functional capacity over and above the effects of the TAVI procedure itself.

## 3. CPET and Transcatheter Treatment of Severe Mitral Regurgitation

Organic MR is caused by a primary abnormality of one or more elements of the valve apparatus [1]. Moreover, in patients with HF and dilated left ventricle, MR can develop due to geometric displacement in papillary muscles and chordae tendineae, which impairs leaflet coaptation [25]. This functional MR increases the severity of volume overload and is associated with reduced quality of life, repeated hospitalizations for HF, and poor survival [26]. Guideline-directed medical therapy and cardiac resynchronization therapy can deliver symptomatic relief, improve left ventricular function, and, in some patients, reduce the severity of MR [26]. Neither surgical valve replacement nor surgical valve repair has been shown to reduce hospitalization rates or death in patients with functional MR, and both procedures carry a significant risk of complications [1]. As a result, most patients with HF and functional MR are treated conservatively and often have few therapeutic alternatives. For those at high surgical risk, transcatheter mitral valve interventions represent an emerging treatment option [27] without relevant differences in outcome between the two MR aetiologies [28, 29]. Due to the complexity of the mitral valve anatomy, different techniques have been developed to target specific components of MR. Mitral transcatheter edge-to-edge repair (TEER) utilizes a clip to bring the valve leaflets closer together, effectively treating severe MR and avoiding the risks associated with open surgery [30]. The COAPT trial [31] enrolled 614 patients with moderate-to-severe or severe functional MR and HF who were randomized 1:1 to receive either TEER and medical therapy (device group) or medical therapy alone (control group). The annualized rates of all-cause hospitalization for HF within 24 months were 35.8% per patient–year in the device group vs. 67.9% per patient–year in the control group (hazard ratio (HR) 0.53; 95% CI 0.40 to 0.70; *p* < 0.001). Any cause of death at 24 months occurred in 29.1% of patients in the device arm compared to 46.1% in the control arm (HR 0.62; 95% CI 0.46 to 0.82; *p* < 0.001). The quality of life was measured using the KCCQ score (on a scale of 0 to 100, with a higher score indicating improved quality of life) and the 6MWT (with longer distances indicating better functional capacity). At 12 months, the KCCQ score changed by a mean (± SD) of +12.5 ± 1.8 points in the device group vs. –3.6 ± 1.9 points in the control group (*p* < 0.001), and the 6MWT changed by a mean (± SD) of –2.2 ± 9.1 meters vs. –60.2 ± 9.0 meters, respectively (*p* < 0.001). However, subjective symptoms, functional class, and 6MWT assessment may be confounded by other variables [32], such as a sedentary lifestyle or self-imposed restrictions on physical activity. Therefore, the role of transcatheter mitral repair in objectifying the improvement in functional capacity remains to be fully elucidated. Benito-González *et al*. [33] presented a single-center prospective registry on TEER with MitraClip implantation in 11 patients having functional MR and HF, which focused on functional outcomes assessed by CPET. At the 6-month follow-up, the VO_2_ increased from 9.8 mL/min/kg to 13.5 mL/min/kg (*p* = 0.033); VO_2_ at AT increased from 510 mL/min to 850 mL/min (*p* = 0.033); O_2_ pulse increased from 7.2 mL/beat to 8.3 mL/beat (*p* = 0.013); VE/VCO_2_ slope increased from 30 to 31.5 (*p* = NS); oxygen uptake efficiency slope (OUES) increased from 1035 to 1135 (*p* = 0.033). Moreover, Koh *et al*. [34] presented a single-center retrospective experience using MitraClip for direct mitral leaflet repair focused on functional outcomes assessed by CPET. After a median of 203 days, all patients (N = 7) showed improvement in cardiopulmonary capacity: peak VO_2_ from 14.3 mL/min/kg to 17.8 mL/min/kg (+25%), VO_2_ at AT from 792 mL/min to 887 mL/min (+12%), O_2_ pulse from 6.9 mL/beat to 7.8 mL/beat (+13%), and VE/VCO_2_ slope from 35.5 to 33.4 (–6%). Finally, Vignati *et al*. [35] evaluated changes in peak VO_2_ and cardiac output (CO) after successful mitral TEER in a single-center study on 145 patients with severe organic and functional MR who underwent non-invasive CO measurement (through inert gas rebreathing, Innocor Rebreathing System) and CPET examination before intervention and at a 6-month follow-up. Peak exercise CO increased significantly (from 5.9 ± 2.0 L/min to 6.5 ± 1.8, *p* < 0.001), with a parallel reduction in arteriovenous O_2_ content difference [ΔC(a-v)O_2_] (from 16.4 ± 4.0 to 15.2 ± 4.1, *p *= 0.009), whereas peak VO_2_ remained unchanged (from 936 ± 260 mL/min to 962 ± 241, *p* = 0.24). The authors suggest that the divergence between peak CO improvement and unchanged peak VO_2_ after mitral TEER is difficult to understand and deserves a physiologically based discussion. In patients with severe HF, as those with functional MR enrolled in Vignati’s investigation [35], low resting CO was compensated by increased ΔC(a-v)O_2_. In fact, resting ΔC(a-v)O_2_ values in the study population were almost twice those normally observed in healthy individuals, showing that patients had to rely on compensatory mechanisms of low CO to preserve aerobic metabolism even at rest. As CO improved, ΔC(a-v)O_2_ decreased, suggesting that, since VO_2_ was constant, there was a decrease in O_2_ extraction in the peripheral tissues and probably a redistribution in blood flow from high to low extraction tissues. This enabled a more “physiological” blood flow redistribution and O_2_ extraction response. Indeed, restricting the assessment to VO_2_ does not allow for a proper evaluation of therapeutic interventions, with a significant change in the delivery of blood flow during exercise [36]. Table 1 (Ref. [33, 34, 35, 37, 38]) indicates, in detail, the CPET results among patients undergoing mitral TEER in the aforementioned investigations.

**Table 1.** **Cardiopulmonary exercise testing characteristics in patients undergoing transcatheter mitral and tricuspid valve repair**.

Study (year)	Type of study	No. of patients	Follow-up, months	CPET-derived variables											
				Pre-TEER				Post-TEER				Change			
				Peak VO_2_, mL/min/kg or (mL/min)	VO_2_ at AT, mL/min	O_2_ pulse, mL/beat	VE/VCO_2_ slope	Peak VO_2_, mL/min/kg or (mL/min)	VO_2_ at AT, mL/min	O_2_ pulse, mL/beat	VE/VCO_2_ slope	Peak VO_2_, mL/min/kg or (mL/min)	VO_2_ at AT, mL/min	O_2_ pulse, mL/beat	VE/VCO_2_ slope
Mitral regurgitation															
Benito-González *et al*. (2019) [33]	Prospective	11	6	9.8	510	7.2	30	13.5	850	8.3	31.5	+3.7	+340	+ 1.1	+1.5
Vignati *et al*. (2021) [35]	Prospective	66	6	(936)	708	∖	34.2	(962)	740	∖	33.9	(+26)	+32	∖	–0.3
Koh (2023) [34]	Retrospective	7	7 ± 1	14.3	792	6.9	35.5	17.8	887	7.8	33.4	+3.5	+95	+0.9	–2.1
Tricuspid regurgitation															
Volz *et al*. (2022) [37]	Retrospective	11	3	9.5	639	∖	39	11.4	749	∖	38	+1.9	+110	∖	–1
Cumitini *et al*. (2024) [38]	Case report	1	1	12	610	6.2	31.8	14.4	750	8.2	31.8	+2.4	+140	+2	0

All CPET values (except for the case report) are expressed as the mean or median. CPET, cardiopulmonary exercise testing; TEER, transcatheter edge-to-edge repair; VE/VCO_2_ slope, minute ventilation/carbon dioxide production slope; VO_2_, oxygen consumption; VO_2_ at AT, oxygen uptake at anaerobic threshold.

## 4. CPET and Transcatheter Treatment of Severe Tricuspid Regurgitation

TR is a widespread valve disease in Western countries, with a prevalence of >60% [39]. Severe or greater TR is associated with an impaired prognosis, with an estimated 5-year survival rate of 30% compared to patients without relevant TR. The recent acknowledgment that TR is associated with independent prognostic implications on subsequent clinical outcomes has focused on various treatment approaches [40]. Here, medical treatment does not affect survival. Surgical management of isolated TR is often challenging due to patient comorbidities that increase the postoperative risk, such as right ventricular failure or hepatorenal syndrome, as a result of chronic venous congestion [41]. For patients with severe TR, TEER has proven to be a safe and potentially successful treatment [42, 43]. This procedure uses a transvenous approach to approximate the tricuspid valve leaflets by positioning a clip and holding the leaflets together, reducing the regurgitation without needing cardiopulmonary bypass or cardiac surgery [43]. The recent TRILUMINATE trial [42] enrolled 350 patients with symptomatic severe TR randomized 1:1 to receive TEER (device group) or medical therapy (control group). The primary endpoint was a hierarchical composite of any cause of death or tricuspid valve surgery, hospitalization for HF, and improvement in quality of life, as measured by KCCQ (defined as a rise of at least 15 points) at 1-year follow-up. There was no difference between the two groups in the incidence of death, tricuspid valve surgery, or rates of hospitalization for HF. The KCCQ quality of life score changed by a mean (± SD) of +12.3 ± 1.8 points in the device group vs. +0.6 ± 1.8 points in the control group (*p* < 0.001). However, the role of transcatheter tricuspid repair in objectifying the improvement in functional capacity remains to be fully clarified. Currently, there is limited evidence regarding the effect of this procedure on the amelioration of CPET parameters. Volz *et al*. [37] presented a single-center, retrospective experience using the PASCAL Ace device. After the intervention, all patients (N = 11) showed at 3 months a significant improvement of VO_2_ max (9.5 ± 2.8 mL/kg/min vs. 11.4 ± 3.4 mL/kg/min at baseline, *p* = 0.006). Additionally, peak VO_2_ increased from 703 ± 175 to 826 ± 198 mL/min (*p* = 0.004), VO_2_ max percent predicted from 42 ± 12% to 50 ± 15% (*p* = 0.004), and O_2_ pulse percent predicted from 67 ± 21% to 81 ± 25% (*p* = 0.011). The other CPET-related variables showed no significant post-procedural changes. In a recent case report [38], a patient who underwent TEER for severe TR showed an early significant improvement one month after the intervention in peak VO_2_ (12.0 mL/kg/min vs. 14.4 mL/kg/min, 82% vs. 102% predicted) and O_2_ pulse (6.2 mL/beat vs. 8.2 mL/beat, 66% vs. 85% predicted) at CPET. Table 1 shows CPET results in patients undergoing tricuspid TEER from available studies.

## 5. Future Directions

In patients with HF, the presence of VHD has a prognostic significance, while new transcatheter treatment options have emerged. In this context, CPET is accurate for evaluating and managing various cardiopulmonary symptoms and clinical conditions. Therefore, it may be a useful tool for several purposes. For example, if baseline CPET parameters are anomalous, the test could discriminate borderline patients for performing or not performing the intervention. It is also helpful to guide clinical management and follow-up, especially in patients with predefined poorer variables. After percutaneous procedures, CPET may provide objective evidence documenting changes in the cardiorespiratory endurance of the patient (Table 2). By objectifying an improvement in functional capacity, CPET may increase the strength of guideline recommendations for percutaneous valve repair. Since published studies derive from small, non-randomized series, it is important to recognize that their results are only hypothesis-generating and require further confirmation in adequately powered prospective investigations.

**Table 2.** **Potential role of cardiopulmonary exercise testing in patients undergoing transcatheter valve procedures**.

Assess functional capacity and exercise intolerance
Recognize different causes of exercise limitation
Discriminate borderline patients for the intervention
Contribute to clinical management, especially for patients with predefined poorer values
Evaluate clinical progression during follow-up
Provide objective variables documenting changes in cardiorespiratory endurance after the procedure

Recently, numerous other diagnostic tools have been identified, in addition to CPET (also called “complex CPET” [6]), to provide additional clinical and pathophysiological data that could be used in patients undergoing transcatheter valve procedures [6]. Non-invasive CO measurement has been proposed, and two approaches during the exercise are the most recognized: the Physioflow technique and the inert gas rebreathing technique. The former is based on thoracic bioimpedance measurements and the latter utilizes a blood-soluble and a blood-insoluble inert gas. The concentration of the blood-soluble gas drops during rebreathing at a rate related to the pulmonary blood flow, while the insoluble gas establishes the lung volume [6, 44, 45]. Both tools allow VO_2_ to be split into its two components, CO and ΔC(a-v)O_2_, according to the Fick principle. Therefore, by separating the cardiac and peripheral roles, it is possible to explain the cause of VO_2_ limitation [6, 45].

Furthermore, near-infrared spectroscopy quantitatively evaluates oxygenated and deoxygenated hemoglobin [46], thereby representing a promising tool for better understanding the role of O_2_ delivery to the working muscle and its use [6, 47]. Arterial blood sampling has a well-defined clinical role in evaluating the ratio of death to tidal volume during the exercise, because it can only be reliably assessed by directly measuring arterial CO_2_ partial pressure [48]. Vignati *et al*. [35] suggested that the restriction of the assessment to VO_2_ only does not allow an accurate evaluation of the impact of therapeutic interventions, such as TEER, cardiac resynchronization therapy, or training, because of the significant change in blood flow delivery during the exercise [36, 49]. Thus, all the aforementioned techniques can be combined with CPET in patients with VHD to better define the pathophysiology of exercise in this setting.

## 6. Conclusions

Although limited by the small number of patients enrolled in the studies and the lack of powered randomized trials, transcatheter valve procedures appear to be associated with improved cardiopulmonary performance. In this setting, detecting such functional improvement may be clinically significant and have a prognostic relevance. Given its objective, specific, and unique information, CPET can emerge as a promising tool for addressing this issue.
